# Supplementary material for: Relationships between neuropsychological and antisaccade measures in multiple sclerosis patients
Source: PeerJ. 2018 Oct 4;6:e5737. doi: 10.7717/peerj.5737 (PMC6174870; doi:10.7717/peerj.5737)
Supplement: Supplemental Information 2 [file peerj-06-5737-s002.pdf]

Latency analysis

R code

```
setwd("~/Desktop")
data = read.csv2 (file ="nome1.csv" , sep = "," , dec=".")
library(nlme)
library(lsmeans)
library(multcomp)

# model for latency
model = lme(out_latency ~group*factor(sac_type) + factor(trial_direction) , random =
~1|id_aaa , data = data)
summary(model)
```

R output

```
[illegible]
> # model for latency
> model = lme(out_latency ~group*factor(sac_type) + factor(trial_direction) , random = ~1|id_aaa , data =
data)
> summary(model)
Linear mixed-effects model fit by REML
 Data: data
       AIC      BIC    logLik
  25899.58 25951.76 -12940.79

Random effects:
 Formula: ~1 | id_aaa
        (Intercept) Residual
StdDev:     35.9196 46.52142

Fixed effects: out_latency ~ group * factor(sac_type) + factor(trial_direction)
                            Value Std.Error   DF   t-value p-value
(Intercept)             209.32477  7.391202 2362 28.320801  0.0000
groupM                    9.73365 10.070463   74  0.966554  0.3369
factor(sac_type)2       -50.59558  6.024032 2362 -8.398957  0.0000
factor(sac_type)3        76.35367  4.719172 2362 16.179463  0.0000
factor(trial_direction)2 -10.01256  1.902614 2362 -5.262529  0.0000
groupM:factor(sac_type)2   2.81405  7.790842 2362  0.361200  0.7180
groupM:factor(sac_type)3  34.41149  6.283663 2362  5.476343  0.0000
 Correlation:
                         (Intr) groupM fctr(s_)2 fc(_)3 fctr(t_)2 gM:(_)2
groupM                   -0.721
factor(sac_type)2        -0.425  0.311
factor(sac_type)3        -0.570  0.416  0.663
factor(trial_direction)2 -0.143  0.017  0.013     0.028
groupM:factor(sac_type)2  0.327 -0.403 -0.773    -0.513  0.000
groupM:factor(sac_type)3  0.430 -0.537 -0.498    -0.751 -0.030     0.646

Standardized Within-Group Residuals:
       Min         Q1        Med         Q3        Max
-4.2917151 -0.6400065 -0.1147621  0.5551761  4.4036597

Number of Observations: 2443
Number of Groups: 76
```

saccade type
1 = pro saccade
2 = corrective
3 = antisaccade
C = control
M = Multiple Sclerosis

Latency pairwise comparisons

saccade type: 1 = pro saccade; 2 = corrective
3 = antisaccade; C = control; M = Multiple Sclerosis

```
> # comparing by group*saccade type
> summary(glht(model, lsm(pairwise ~ group*sac_type)))
Note: df set[illegible]

	 Simultaneous Tests for General Linear Hypotheses

Fit: lme.formula(fixed = out_latency ~ group * factor(sac_type) +
    factor(trial_direction), data = data, random = ~1 | id_aaa)

Linear Hypotheses:
                 Estimate Std. Error t value Pr(>|t|)
C,1 - M,1 == 0     -9.734     10.070  -0.967  0.91058
C,1 - C,2 == 0     50.596      6.024   8.399  < 1e-04 ***
C,1 - M,2 == 0     38.048     10.021   3.797  0.00159 **
C,1 - C,3 == 0    -76.354      4.719 -16.179  < 1e-04 ***
C,1 - M,3 == 0   -120.499      9.498 -12.686  < 1e-04 ***
M,1 - C,2 == 0     60.329      9.998   6.034  < 1e-04 ***
M,1 - M,2 == 0     47.782      4.942   9.669  < 1e-04 ***
M,1 - C,3 == 0    -66.620      9.173  -7.263  < 1e-04 ***
M,1 - M,3 == 0   -110.765      4.147 -26.709  < 1e-04 ***
C,2 - M,2 == 0    -12.548      9.947  -1.261  0.76816
C,2 - C,3 == 0   -126.949      4.566 -27.804  < 1e-04 ***
C,2 - M,3 == 0   -171.094      9.422 -18.159  < 1e-04 ***
M,2 - C,3 == 0   -114.402      9.117 -12.548  < 1e-04 ***
M,2 - M,3 == 0   -158.547      4.011 -39.526  < 1e-04 ***
C,3 - M,3 == 0    -44.145      8.542  -5.168  < 1e-04 ***
---
Signif. codes:  0 ‘***’ 0.001 ‘**’ 0.01 ‘*’ 0.05 ‘.’ 0.1 ‘ ’ 1
(Adjusted p values reported -- single-step method)
```

Peak-velocity analysis

#model for peak-velocity
model = lme(out_peak_vel ~group*factor(sac_type) + factor(trial_direction) , random = ~1|id_aaa , data = data)
summary(model)

```
> #model for peak-velocity
> model = lme(out_peak_vel ~group*factor(sac_type) + factor(trial_direction) , random = ~1|id_aaa , data =
data)
> summary(model)
Linear mixed-effects model fit by REML
 Data: data
       AIC      BIC    logLik
  27439.32 27491.51 -13710.66

Random effects:
 Formula: ~1 | id_aaa
        (Intercept) Residual
StdDev:    60.69536 63.41535

Fixed effects: out_peak_vel ~ group * factor(sac_type) + factor(trial_direction)
                              Value Std.Error   DF   t-value p-value
(Intercept)               267.41917 11.636172 2362 22.981713  0.0000
groupM                     -3.11578 16.008029   74 -0.194639  0.8462
factor(sac_type)2          59.73596  8.212576 2362  7.273718  0.0000
factor(sac_type)3           0.51402  6.437047 2362  0.079853  0.9364
factor(trial_direction)2  -12.55383  2.594122 2362 -4.839338  0.0000
groupM:factor(sac_type)2    2.82176 10.621786 2362  0.265658  0.7905
groupM:factor(sac_type)3    6.78688  8.574147 2362  0.791552  0.4287
 Correlation:
                         (Intr) groupM fctr(s_)2 fc(_)3 fctr(t_)2 gM:(_)2
groupM                   -0.718
factor(sac_type)2        -0.368  0.267
factor(sac_type)3        -0.494  0.357  0.663
factor(trial_direction)2 -0.124  0.015  0.013     0.028
groupM:factor(sac_type)2  0.283 -0.345 -0.773    -0.512  0.000
groupM:factor(sac_type)3  0.372 -0.461 -0.498    -0.751 -0.031     0.645

Standardized Within-Group Residuals:
        Min          Q1         Med          Q3         Max
-4.82559829 -0.49353432 -0.02731636  0.40808920 10.12643751

Number of Observations: 2443
Number of Groups: 76
>
```

saccade type
1 = pro saccade
2 = corrective
3 = antisaccade
C = control
M = Multiple Sclerosis

Peak-velocity pairwise comparisons

```
# comparing by group*saccade type
summary(glht(model, lsm(pairwise ~ group*sac_type)))
```

```
> # comparing by group*saccade type
> summary(glht(model, lsm(pairwise ~ group*sac_type)))
Note: df set to 989

     Simultaneous Tests for General Linear Hypotheses

Fit: lme.formula(fixed = out_peak_vel ~ group * factor(sac_type) +
    factor(trial_direction), data = data, random = ~1 | id_aaa)

Linear Hypotheses:
               Estimate Std. Error t value Pr(>|t|)
C,1 - M,1 == 0    3.116     16.008   0.195  0.99995
C,1 - C,2 == 0  -59.736      8.213  -7.274  < 0.001 ***
C,1 - M,2 == 0  -59.442     15.949  -3.727  0.00195 **
C,1 - C,3 == 0   -0.514      6.437  -0.080  1.00000
C,1 - M,3 == 0   -4.185     15.342  -0.273  0.99972
M,1 - C,2 == 0  -62.852     15.924  -3.947  < 0.001 ***
M,1 - M,2 == 0  -62.558      6.738  -9.284  < 0.001 ***
M,1 - C,3 == 0   -3.630     14.971  -0.242  0.99984
M,1 - M,3 == 0   -7.301      5.661  -1.290  0.74515
C,2 - M,2 == 0    0.294     15.864   0.019  1.00000
C,2 - C,3 == 0   59.222      6.229   9.508  < 0.001 ***
C,2 - M,3 == 0   55.551     15.255   3.641  0.00273 **
M,2 - C,3 == 0   58.928     14.907   3.953  < 0.001 ***
M,2 - M,3 == 0   55.257      5.474  10.093  < 0.001 ***
C,3 - M,3 == 0   -3.671     14.258  -0.257  0.99979
---
Signif. codes:  0 '***' 0.001 '**' 0.01 '*' 0.05 '.' 0.1 ' ' 1
(Adjusted p values reported -- single-step method)
```

saccade type
1 = pro saccade
2 = corrective
3 = antisaccade
C = control
M = Multiple Sclerosis빵
